# Supplementary material for: Hepatocyte Growth Factor/MET Activator Rescues Working Memory Deficits after Repeated Mild Traumatic Brain Injury
Source: Neurotrauma Rep. 2025 Nov 20;6(1):991–8. doi: 10.1177/2689288X251392851 (PMC13629188; doi:10.1177/2689288X251392851)
Supplement: Supplementary file 2 [file sj-docx-2-ntr-10.1177_2689288X251392851.docx]

**Supplementary Figure 1.** Performance in the T-maze delayed alternation task of working memory during Baseline and after repeated sham or mTBI surgeries. 20 trials; 50% is chance performance. A) Performance in the T-maze task for all subjects and (B) separated out by sex. C) Total duration of T-maze testing for all subjects and (D) separated out by sex. #p<0.05, ##p<0.01, ###p<0.001, Tukey's HSD.
